# Heterogeneiety and Cognitive Status Among Older Black Men

**DOI:** 10.1093/geroni/igaf122.1302

**Published:** 2025-12-31

**Authors:** Marino Bruce, Roland Thorpe, Jr., Gillian Marshall, Charles Udo, Kiara Spooner, Bettina Beech

**Affiliations:** University of Houston, Houston, Texas, United States; Johns Hopkins University, Baltimore, Maryland, United States; Dynamic and Innovative Research Solutions, Seattle, Washington, United States; University of Houston, Houston, Texas, United States; University of Houston, Houston, Texas, United States; University of Houston, Houston, Texas, United States

## Abstract

Persistent and cumulative inequality have contributed to disproportionate levels of cognitive impairment and decline among Black men. The association between inequality and cognitive outcomes among this population is deemed complex and recent studies have suggested that heterogeneity can be an important consideration. The purpose of this study was to examine how heterogeneity influences correlates of cognitive impairment among Black men in the Health and Retirement Study (HRS). Data were drawn from participants who completed the Core and Leave Behind Questionnaire items in the 2016 HRS. Any cognitive impairment was the outcome of interest. Income status was the primary contextual variable indicating whether particpants had incomes above or below the national medican income ($50,000). Income status distinctions were observed for every variable in the study. There was a significant difference in any cognitive impairment as the proportion of Black men with incomes less than $50,000 (48.2%) more than doubled the corresponding percentage for their more affluent peers (18.4%). Results from stratified Modified Poisoon Regression models indicated that correlates associated with any cognitive impairment varied by income status. Age (PR = 1.02: CI 1.00 – 1.03) and poor health (PR = 1.43: CI 1.08 – 1.90) were significant for men reporting incomes below the national median while post-secondary education (PR = 0.22: CI: 0.09 – 0.51) and depressive symptoms (PR = 1.19 CI: 1.02 – 1.39) were associated with any cognitive impairment among Black men with incomes over $50,000. Additional studies are needed to advance our understanding how heterogeneity can impact research examining cognitive impairment and decline among Black men.

